# How supervisor-subordinate emotional appraisal ability congruence influences unethical pro-supervisor behavior: the mediating effect of supervisor-subordinate guanxi

**DOI:** 10.1186/s40359-026-04775-0

**Published:** 2026-05-18

**Authors:** ChongRong Huang, ChengYan Li, Luo Deng

**Affiliations:** 1 College of Education, Dongguan University of Technology, Dongguan, China; 2 School of Philosophy, law and politics, Shanghai Normal University, Shanghai, China; 3 College of Psychology, Shanghai Normal University, Shanghai, China

**Keywords:** Emotional Appraisal Ability, Supervisor-Subordinate Guanxi, Unethical Pro-Supervisor Behavior, Response Surface Analysis

## Abstract

Drawing on Person–Environment Fit Theory and Social Exchange Theory, this study examined the effects of supervisor–subordinate congruence in emotional appraisal ability on unethical pro-supervisor behavior and its underlying mechanism. Specifically, the results of a scenario simulation experiment and a multiphasic questionnaire design showed that (1) Congruence in supervisor-subordinate emotional appraisal ability promotes supervisor-subordinate guanxi and unethical pro-supervisor behavior more effectively than situations of incongruence. (2) In congruence situations, high supervisor-high subordinate emotional appraisal ability is more likely to promote supervisor-subordinate guanxi and unethical pro-supervisor behavior than low supervisor-low subordinate emotional appraisal ability. (3) In incongruence situations, a scenario experiment (Study 1) showed that low supervisor - high subordinate emotional appraisal ability is more likely to promote supervisor-subordinate guanxi and unethical pro-supervisor behavior than high supervisor - low subordinate emotional appraisal ability; however, this asymmetric effect on supervisor-subordinate guanxi was not replicated in the questionnaire survey (Study 2). (4) Supervisor-subordinate guanxi mediates between supervisor-subordinate emotional appraisal ability congruence and unethical pro-supervisor behavior. (5) The moderating effect of subordinate’s gender on the congruence of emotional appraisal ability through the mediating effect of supervisor-subordinate guanxi on unethical pro-supervisor behavior is not significant.

## Introduction

In recent years, researchers have observed that employees may engage in unethical behavior not only for self-serving but also for altruistic motives [1–3]. Specifically, employee behavior that violates laws, regulations, or social and moral norms to protect the supervisor’s interests is referred to as Unethical Pro-supervisor Behavior (UPSB) [1]. In the short term, such behavior may yield immediate benefits for the supervisor. However, in the long run, it can lead to substantial costs for the organization. For instance, it may hinder the timely resolution of potentially serious issues and damage the organization’s external reputation [4]. Therefore, it is crucial to address and implement measures to prevent employees from engaging in unethical pro-supervisor behavior.

Unethical pro-supervisor behavior comprises two fundamental components [1–3]. First, by definition, UPSB is inherently unethical, carrying the same moral valence as other forms of unethical conduct. Second, its underlying intention is pro-supervisor, indicating that employees engage in such acts primarily to benefit their supervisor rather than to serve their own interests. Importantly, these actions are neither formally mandated nor explicitly sanctioned by the organization or the supervisor [5, 6]. To further delineate the conceptual boundaries of UPSB, the present study adopts the definition of unethical pro-supervisor behavior advanced by Umphress et al. (2010) [5], which delineates three boundary conditions. First, with regard to its nature, UPSB constitutes employees’ voluntary and intentional acts. It does not stem from accidents, mistakes, or misunderstandings. Second, with respect to motivation, the primary purpose of engaging in UPSB is to protect or benefit one’s supervisor. If a subordinate performs unethical acts solely out of self-interest—and such acts incidentally yield benefits to the supervisor—those actions cannot be classified as UPSB. Finally, in terms of outcomes, although UPSB may appear advantageous in the short term or at the individual supervisor level, it ultimately inflicts harm on the organization over the long term. The negative consequences arising from such behavior must ultimately be borne by the supervisor and the organization at large.

Currently, research on the antecedents of unethical pro-supervisor behavior has focused primarily on two relatively distinct dimensions: individual factors [1] and environmental factors [7]. However, according to person-environment fit theory, individual behavior is a function of the interplay between personal attributes and situational conditions [8]. A subordinate’s behavior is shaped not only by the independent effects of their own attributes and those of the supervisor, but also by the interplay and even the congruence between the two [9, 10]. Hence, it is necessary to examine the influence of supervisor-subordinate fit on the emergence of unethical pro-supervisor behavior. A study published in the *Harvard Business Review* (2019) pointed out that there is a latent influence of supervisors’ emotional traits on their subordinates, further emphasizing that achieving “dynamic resonance” between a supervisor’s and a subordinate’s emotions carries practical significance for shaping both subordinate and supervisor behavior.

Unfortunately, there has not yet been an interpretation of the antecedents of UPSB from the perspective of congruence between individual and supervisor emotional appraisal abilities. To address this gap, the present study explores the factors that contribute to the formation of UPSB by focusing on the congruence between employees’ and supervisors’ emotional appraisal ability. Emotional appraisal ability(EAA) refers to an individual’s ability to perceive and understand others’ emotions as well as to be able to perform appropriate behaviors in work and life by appraising and understanding others’ emotions, which is one of the dimensions of emotional intelligence [11].

The present study selects the congruence of emotional appraisal ability between supervisor and subordinates as an antecedent of unethical pro-supervisor behavior, given the conceptual and logical alignment between this variable and the behavioral mechanism underlying UPSB. Unlike comparatively stable traits such as moral reasoning or personal values, emotional appraisal ability constitutes a form of emotional processing and interpersonal perceptual capacity. Its core function lies in accurately recognizing, understanding, and responding to others’ emotional cues [11–13], as opposed to involving value-based moral judgments. The defining feature of UPSB is that it constitutes altruistic yet unethical conduct driven by interpersonal emotional bonds [1–5]. The occurrence of such behavior does not stem from value congruence or moral identification but rather from subordinates’ acute sensitivity to their supervisors’ emotional needs and their proactive efforts to meet those needs. This behavioral mechanism corresponds closely with the functional essence of emotional appraisal ability. Accordingly, this study positions the supervisor–subordinate EAA congruence as an explanatory factor in accounting for the occurrence of UPSB.

To further clarify the logical pathway between supervisor-subordinate emotion appraisal abilities congruence and unethical pro-supervisor behavior, this study investigates the underlying mechanisms involved. Through a review of the existing literature, we propose that the supervisor-subordinate guanxi may serve as a mediating mechanism. On the one hand, the Individual-Environment Fit Theory posits that similarity in characteristics, such as personality, can foster convergent thinking styles between interacting individuals, thereby diminishing psychological distance and fortifying relational bonds [14–16]. Specifically, congruence in emotion appraisal abilities between supervisors and subordinates can effectively facilitate communication and interaction, thereby cultivating a more robust supervisor-subordinate guanxi. On the other hand, those who develop high-quality relationships with their supervisors are more likely to receive certain privileges and advantages compared to employees with low-quality relationships [17]. According to the reciprocity principle of social exchange theory, employees with high-quality relationships are likely to reciprocate the preferential treatment they receive from their supervisors through their work. For example, they may prioritize protecting their supervisor’s interests, even in situations that conflict with societal morals, by engaging in unethical pro-supervisor behaviors.

Furthermore, does the aforementioned pathway exhibit stability across genders? Brackett et al. (2006) [18]emphasized the importance of considering gender in models concerning emotional intelligence research. Social norms prescribe stronger communal role expectations for women, stressing affiliation, empathy, and emotional maintenance [19, 20]. Such normative pressures lead female employees to engage more intensively in emotional labor within the workplace, actively managing others’ emotions to maintain interpersonal harmony [21, 22].Consequently, when faced with identical emotional cues from their supervisors, female subordinates may devote greater cognitive resources to interpreting those signals. In contrast, male subordinates are bound by social role norms that discourage overt emotional expression [23]. They tend to demonstrate comparatively muted emotional engagement even when possessing high emotional appraisal ability [24].Therefore, this study posits that this pathway may not be invariant across genders: when the subordinate is female, she is more likely to detect the supervisor’s emotional fluctuations with greater acuity and to address the supervisor’s emotional needs more accurately and with greater alacrity. Such attunement facilitates the development of high-quality supervisor–subordinate guanxi, which in turn may heighten the propensity to enact in unethical pro-supervisor behaviors.

In summary, the present study aims to examine the relationship between supervisor-subordinate emotional appraisal ability congruence and unethical pro-supervisor behavior, along with the mechanisms underlying this association. Specifically, based on individual-environment fit theory and social exchange theory, the present study investigates the effects of supervisor-subordinate emotional appraisal ability congruence on unethical pro-supervisor behaviors, and explores the role of supervisor-subordinate guanxi and subordinate gender. The present study contributes to extending the antecedents of unethical pro-supervisor behaviors in organizations and provides practical suggestions for reducing unethical pro-supervisor behaviors from a dual-matching perspective.

### Categories of congruence between supervisors’ and subordinates’ emotional appraisal ability

Emotional appraisal ability may be distinguished by referent into supervisor emotional appraisal ability and subordinate emotional appraisal ability. Supervisor emotional appraisal ability refers to a supervisor’s ability to perceive and interpret the emotions of their subordinates, while subordinate emotional appraisal ability pertains to a subordinate’s ability to perceive and interpret the emotions of their supervisor. Based on the relative levels of this ability exhibited by supervisors and subordinates, four dyadic configurations emerge. Congruence occurs in two scenarios: (1) high supervisor emotional appraisal ability paired with high subordinate emotional appraisal ability, and (2) low supervisor emotional appraisal ability paired with low subordinate emotional appraisal ability. Incongruence likewise encompasses two scenarios: (3) high supervisor emotional appraisal ability paired with low subordinate emotional appraisal ability, and (4) low supervisor emotional appraisal ability paired with high subordinate emotional appraisal ability.

### Congruence of emotional appraisal ability, supervisor-subordinate guanxi, and unethical pro-supervisor behavior

At present, two primary variables are used to describe supervisor-subordinate interactions: “Leader-Member Exchange (LMX)” [17], proposed by Western scholars, and “Supervisor-Subordinate Guanxi (SSG)” [25], introduced by Eastern scholars. LMX emphasizes the overall quality of the exchange relationship in the workplace, representing the formal working relationship between supervisors and subordinates [17]. SSG refers to the personal relationship between supervisors and subordinates through informal social interactions. It extends beyond the formal work relationship to include private and emotional interactions outside the workplace, incorporating elements of social interaction such as dining together and exchanging greetings [25]. As the cultural context of the study is based on the Chinese context, SSG is considered a more culturally appropriate representation of supervisor-subordinate relationships than LMX. Therefore, the variable of Supervisor-Subordinate Guanxi (SSG) was selected for this study.

Based on the perspective of supplementary fit in person-environment theories, individuals who achieve congruence between their needs and the environment’s supplies are more likely to experience positive cognitive states [15, 26, 27]. This suggests that when subordinates and supervisors exhibit high congruence in certain aspects, subordinates are more likely to demonstrate strong adaptability and flexible cognitive states in the given context, ultimately enhancing their work behaviors and overall performance [16]. Specifically, congruence in supervisor–subordinate emotional appraisal ability signals a harmonious alignment of interpersonal attributes, thereby enabling frictionless communication and mutually attuned interactions. Such congruence enhances mutual satisfaction within the relationship [15]. These positive interactions naturally place the subordinate in a state of satisfaction and positivity at work, thus facilitating the cultivation of a high-quality supervisor–subordinate guanxi. Conversely, incongruence between a supervisor’s and subordinate’s emotional appraisal ability may engender misaligned interactional dynamics [28]. Behaviors deemed appropriate by the subordinate may be construed as ill-timed or unsuitable by the supervisor [29]. Under such conditions, the subordinate may be compelled to pursue alternative strategies to accommodate and synchronize with the supervisor. Inevitably, this challenging interaction process leads to low satisfaction with the relationship on both sides, which hinders the development of a constructive supervisor-subordinate relationship. Based on this reasoning, the following hypothesis is proposed:

- H1a: Congruence in supervisor-subordinate emotional appraisal ability is more conducive to fostering supervisor–subordinate guanxi than incongruence.

Congruence in emotional appraisal ability between supervisors and subordinates reflects a high degree of cognitive congruence in how both parties interpret and respond to emotional cues [26, 27]. This congruence creates a harmonious micro social context where both sides can efficiently identify and understand each other’s implicit emotional signals, especially those related to unspoken intentions [30, 31]. Such emotionally based consensus may, to some extent, lead to unethical pro-supervisor behaviors. The reason is that a high level of congruence reduces the threshold for engaging in unethical behaviors. In this kind of matched relationship, supervisors do not need to express unethical intentions directly. Subordinates with strong emotional abilities can perceive subtle emotional expressions and translate them into actions [32]. This form of unspoken emotional coordination furnishes the conditions necessary for unethical behavior to arise. In contrast, when supervisors and subordinates differ in emotional appraisal ability, their emotional interpretation systems become misaligned [28, 29]. This incongruence increases the difficulty for subordinates to interpret and act on ambiguous intentions in an unethical way, which in turn inhibits the occurrence of UPSB. Therefore, the following hypotheses are proposed:

- H1b: Congruence in supervisor–subordinate emotional appraisal ability is more conducive to promoting subordinates’ unethical pro-supervisor behavior than incongruence.

According to the principle of individual-environmental supply-demand complementarity matching, congruence between high supervisor emotion appraisal ability and high subordinate emotion appraisal ability enables both parties to better understand each other’s emotional changes and cognitive tendencies. This congruence fosters an “emotional consensus,” facilitating smoother interactions and deeper mutual understanding [33, 34]. For subordinates, supervisors with high emotional appraisal ability can effectively detect and comprehend their subordinates’ emotional changes and needs, providing timely care and assistance when required [11, 35]. In addition, such supervisors adopt socialization strategies that align with their subordinates’ expectations, enhancing their subordinates’ work experiences [36]. Such behaviors reduce the psychological distance between supervisors and subordinates, which is conducive to the establishment of constructive supervisor-subordinate relationships. For supervisors, subordinates with high emotional appraisal ability can accurately perceive and interpret changes in their supervisors’ emotions and needs, adapting their actions to assist supervisors in achieving their objectives [37]. This adaptability and responsiveness make such subordinates more likely to gain the trust and support of their supervisors. The resulting effective interactions foster psychological empathy between supervisors and subordinates [38], which in turn facilitates the development of high-quality supervisor-subordinate relationships.

On the contrary, when both supervisor and subordinate exhibit low emotional appraisal ability, the supervisor is unable to accurately discern the subordinate’s emotional shifts. Likewise, the subordinate fails to detect the supervisor’s emotional fluctuations. Such interactions result in inefficient communication patterns that fail to satisfy the mutual compatibility requirement of the supplementary fit principle [28]. Within this context, the supervisor and subordinate are in a state of ambiguity regarding each other’s behavioral intentions and cognitive tendencies [29]. As a result, they fail to achieve psychological resonance, making it difficult to establish a high-quality supervisor-subordinate relationship. Furthermore, previous studies have also shown that when there is low efficiency in supervisor-subordinate congruence, it is more likely to result in a lower sense of belonging for the subordinate [39]. In summary, the following hypotheses are proposed:

- H2a: Under conditions of congruent emotional appraisal ability, the combination of high supervisor-high subordinate emotion appraisal ability will promote supervisor-subordinate guanxi more effectively than the combination of low supervisor-low subordinate emotion appraisal ability.

In the high–high congruence condition, both supervisors and subordinates possess a high level of emotional appraisal ability. This congruence creates an interaction system that is efficient and low in friction [33, 34]. Such a system not only facilitates smooth communication but may also act as a driving force that promotes unethical pro-supervisor behaviors. This may occur because an emotionally attuned relationship provides conducive conditions for moral disengagement [40, 41]. When subordinates’ behaviors arise from an empathetic response to the supervisor’s implicit emotional needs rather than obedience to explicit instructions, the mechanism of moral justification is more likely to be activated [42]. Through this mechanism, subordinates cognitively reconstruct unethical pro-supervisor behaviors as positive acts aimed at relieving the supervisor’s burden. Consequently, their perception of immorality is weakened, which further increases their tendency to engage in additional unethical pro-supervisor behaviors.

In contrast, in the low–low congruence condition, both the supervisors and the subordinates exhibit diminished capacities for emotional recognition and responsiveness. Their interactions are often characterized by emotional ambiguity and communication barriers [12, 43]. This low degree of congruence weakens emotional resonance [44], making it difficult for subordinates to accurately understand the supervisor’s emotional intentions. It also deprives them of the psychological conditions required to reinterpret unethical requests as pro-supervisor behaviors. Accordingly, the likelihood of engaging in UPSB is reduced. The following hypotheses are proposed:

- H2b: Under conditions of congruent emotional appraisal ability, the combination of high supervisor-high subordinate emotion appraisal ability will promote unethical pro-supervisor behavior more effectively than the combination of low supervisor-low subordinate emotion appraisal ability.

### Incongruence of emotional appraisal ability, supervisor-subordinate guanxi, and unethical pro-supervisor behavior

Under conditions of incongruence between supervisor emotion appraisal ability and subordinate emotion appraisal ability, the present study proposes that the combination of low supervisor-high subordinate emotion appraisal ability promotes supervisor-subordinate guanxi and unethical pro-supervisor behaviors to a greater extent than the combination of high supervisor-low subordinate emotion appraisal ability.

Specifically, in the incongruent scenario characterized by low supervisor–high subordinate emotion appraisal ability, the supervisor may struggle to understand and perceive the subordinate’s emotional changes. However, the subordinate’s ability to understand and observe the supervisor’s emotional cues allows them to empathize with the supervisor’s situation and anticipate their needs [13, 45]. By adopting the supervisor’s vantage point, subordinates can adjust their behavior to align with the supervisor’s expectations. For example, when the supervisor assigns tasks, the subordinate can proactively accept and efficiently complete them. Beyond formal working hours, they may also engage in more effective communication with the supervisor and remain responsive to the supervisor’s requirements. As a result, the subordinate and supervisor are able to sustain harmonious interactions, thereby fostering a facilitative relational climate.

On the contrary, under conditions of high supervisor-low subordinate emotional appraisal ability incongruence, supervisors are able to perceive their subordinates’ emotional changes and adopt different behavioral strategies [34]. However, the subordinate fails to apprehend the supervisor’s behavioral intentions and expectations, making it difficult for them to respond promptly and accurately. Over time, communication between the supervisor and the subordinate gradually decreases. Their relationship becomes confined to a formal work relationship, with little development in their personal relationship outside of work. As a result, the quality of the supervisor-subordinate guanxi declines. Additionally, research has shown that this type of mismatch tends to lead to the supervisor setting high role expectations for the subordinate [46]. That is, when the supervisor’s high role expectations are not met, it hinders the further development of their relationship.

Based on the above, the following hypotheses are proposed:

- H3a: Under conditions of incongruence in emotional appraisal ability, the combination of low supervisor–high subordinate emotion appraisal ability is more likely to enhance the supervisor-subordinate guanxi than the combination of high supervisor–low subordinate emotion appraisal ability.

Under the incongruent condition of low supervisor–high subordinate emotion appraisal ability, the subordinate’s high emotional appraisal ability can effectively compensate for the supervisor’s low level of emotional appraisal ability, thereby facilitating the emergence of unethical pro-supervisor behaviors (UPSB). Specifically, supervisors with low emotional appraisal ability often have difficulty perceiving emotional cues accurately and expressing their needs clearly [45]. However, subordinates with high emotional appraisal ability are positioned to leverage their acute emotional sensitivity to precisely apprehend the supervisor’s implicit and unarticulated demands [11]. This compensatory matching between a supervisor’s ambiguous needs and a subordinate’s precise emotional insight empowers the subordinate to proactively accommodate the supervisor’s emotional and instrumental needs, which in turn promotes the emergence of UPSB.

In contrast, under the incongruent condition of high supervisor–low subordinate emotion appraisal ability, the supervisor’s high emotional appraisal ability and the subordinate’s low emotional appraisal ability cannot form an effective complementarity, which may inhibit the occurrence of unethical pro-supervisor behaviors. This is attributable to the fact that supervisors with high emotional appraisal ability can accurately perceive the subordinate’s emotional states and behavioral intentions, whereas subordinates with low emotional appraisal ability fail to identify the supervisor’s emotional cues and underlying concerns [47]. Consequently, the interaction between the two parties becomes marked by pronounced information asymmetry. Moreover, owing to their constrained capacity, subordinates lack the requisite sensitivity to gauge the supervisor’s implicit demands and may fear negative consequences caused by behavioral deviations [48]. This further reduces their willingness to engage in UPSB. Therefore, the following hypotheses are proposed:

- H3b: Under conditions of incongruence in emotional appraisal ability, the combination of low supervisor–high subordinate emotion appraisal ability is more likely to promote unethical pro-supervisor behavior than the combination of high supervisor–low subordinate emotion appraisal ability.

### The mediating role of supervisor–subordinate guanxi

Within the Chinese cultural, interactions between supervisors and subordinates extend beyond formal work relationships, often giving rise to informal and particularistic social ties, referred to as supervisor-subordinate guanxi [25]. Drawing on social exchange theory, unethical pro-supervisor behavior is likely a reflection of subordinates’ reciprocal responses toward their supervisors.

Firstly, subordinates with high-quality SSG (Supervisor-Subordinate Guanxi) are inclined to receive greater care and consideration from their supervisors, which fosters a sense of obligation to reciprocate [2]. This sense of obligation triggers their protection and support of the supervisor’s interests, ultimately resulting in unethical pro-supervisor behavior as a form of reciprocation [49].

Secondly, in the Chinese context, the culture of “reciprocity” typically unfolds within asymmetrical exchanges between higher- and lower-status actors [50]. Such asymmetry may impel subordinates embedded in high-quality SSG to protect the supervisor’s interests as much as possible, even when such actions deviate from social norms and ethical standards.

Moreover, empirical research has shown that emotions can influence behavior by triggering cognitive changes [51, 52]. In other words, subordinates with high emotion appraisal ability may affect their perception of the supervisor-subordinate guanxi, which in turn may influence the likelihood of engaging in unethical pro-supervisor behavior.

Lastly, many studies have confirmed that high-quality supervisor-subordinate relationships can promote unethical pro-supervisor behavior [50, 53]. Therefore, this study proposes the following hypothesis:

- H4:Supervisor-subordinate guanxi mediates the relationship between supervisor-subordinate emotional appraisal ability congruence and unethical pro-supervisor behavior.

### The moderating role of subordinate gender

Based on gender differences in emotional intelligence and social role expectations [54, 55], we suggest that gender differences may play a moderating role in the above pathway.

Brackett et al. (2006) [18] emphasized that gender should be incorporated into models when examining emotional intelligence. Social norms assign women stronger communal role expectations that emphasize affiliation, care, and emotional maintenance [19, 20]. These expectations are internalized as role pressures, which make women more likely to engage in emotional labor in the workplace, actively regulating both their own and others’ emotions to maintain interpersonal harmony [21, 22]. Therefore, even when confronted with same emotional cues from supervisors, female subordinates may devote more cognitive resources to interpreting these signals and show stronger behavioral motivation to respond, in order to meet social expectations and strengthen the relationship. Empirical evidence indicates that, compared with men, women are generally perceived as being more affiliative both at work and in daily life [20]. Meta-analytic findings on emotional intelligence further reveal that women exhibit significantly higher levels of emotional intelligence than men, showing stronger emotional perception and emotional regulation abilities [56]. By contrast, male subordinates may be circumscribed by social role norms that discourage overt emotional expression [23]. Even when they possess high emotional appraisal ability, they tend to invest and receive fewer emotional resources in the context of equivalent interactions [24].

Therefore, the present study proposes that when the subordinate is female, they are more likely to sensitively perceive the supervisor’s emotional changes and provide timely responses accordingly. Such effective interactions facilitate the establishment of conductive supervisor-subordinate guanxi, which in turn may lead subordinates to engage in more unethical pro-supervisor behavior. The present study proposes the following hypothesis:

- H5: Congruence in emotional appraisal ability exerts a stronger positive effect on supervisor–subordinate guanxi when the subordinate’s gender is female, which in turn amplifies the subordinate’s unethical pro-supervisor behavior.

**Fig. 1:**
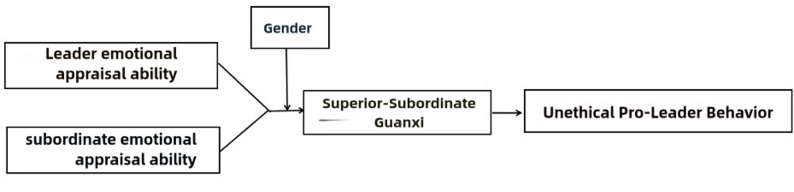
Theoretical model

In summary, the research model constructed of the study is shown in Fig. 1.

## Study 1: The matching effect of congruence in emotional appraisal ability between supervisors and subordinates (scenario experiment)

### Method

#### Participants

Before the study began, the requisite sample size was determined through a statistical power analysis using G*Power 3.1. The analysis indicated a total sample size of 73 was required, based on an assumed effect size of 0.4, an alpha level (α) of 0.05, and a statistical test power of 0.8. Data was collected using online platforms called Credamo and Wenjuanxing.

Participants were recruited through a convenient sampling method. Before data collection began, all participants were informed about the purpose and procedure of the study and were provided with an informed consent form. Each participant received a survey link hosted on a secure online platform. Participants were informed that their participation was voluntary, confidential, and anonymous. They were also told that they could withdraw from the study at any time if they chose not to continue. The inclusion criteria for participation were as follows: (1) being an adults aged 18 years or older; (2) current employment within an organization; and (3) possessing a minimum of 0.6 months of work experience. Ethical approval for the study was obtained from the institutional review board.

An initial pool of 173 responses was collected. However, 13 responses were excluded from the final analysis due to non-compliance with the predetermined data screening criteria. The criteria for data screening included patterned responding, failure to pass attention checks, and a total response time of less than 100 s. Consequently, 160 valid responses were retained for analysis. Among these participants, 63 were male and 97 were female. Their ages ranged from 18 to 51 years, with an mean age of 31.6 years (SD = 7.34). The participants were geographically diverse, hailing from several Chinese cities, including Guangzhou, Shenzhen, Nanjing, Shanghai, Suzhou, and Yangzhou. Regarding educational background, 2.5% reported a high school degree or lower, 10% held an associate degree, 75.6% held a bachelor’s degree, and 11.9% held a master’s degree or above. Missing data were addressed using the Expectation-Maximization algorithm within the maximum likelihood estimation framework.

#### Experimental design and procedures

A 2 (supervisor emotional appraisal ability: high vs. low) × 2 (subordinate emotional appraisal ability: high vs. low) between-subjects experimental design was employed. The dependent variable was unethical pro-supervisor behavior. A scenario simulation experiment was conducted, with the scenario set in a company that manufactures automotive parts. Participants assumed the role of an ordinary employee in the company’s marketing department, with a direct supervisor, Manager Yang, who heads the marketing department.

The experimental procedure was as follows: First, participants completed the Employee Emotional Appraisal Ability Questionnaire. Next, participants were presented with a description of Manager Yang’s emotional appraisal ability, as well as a scenario involving recent unethical behavior. Afterward, participants completed a manipulation check on the supervisor’s emotional appraisal ability and a questionnaire assessing unethical pro-supervisor behavior. Finally, they filled out the Machiavellianism questionnaire, the social desirability scale, and demographic information.

#### Experimental manipulation and measurement of variables

All research variables were measured using well-established scales. Following the translation and back-translation procedure recommended by Brislin (1980) [57], the original English scales were translated into Chinese to ensure linguistic and conceptual equivalence.

##### Supervisor emotional appraisal ability manipulation

The manipulation materials for the supervisor’s emotional appraisal ability were adapted from the study by Li & Luo(2020) [31]. The specific materials are as follows:

- High supervisor emotional appraisal ability condition: “You work in a small company that manufactures automotive mechanical components. Your supervisor is Manager Yang. He shows great concern for you and the team. He often perceives emotional changes in team members and offers appropriate support. He is a supervisor with high emotional intelligence and is good at observing employees’ emotions. He also understands your emotions and is sensitive to your emotional feelings. You get along very well.”
- Low supervisor emotional appraisal ability condition: “You work at a small company that manufactures automotive mechanical components. Your supervisor is Manager Yang. He primarily focuses on your work progress and outcomes, paying little attention to your emotions. He is not sensitive to how you feel and rarely perceives emotional changes. He is a supervisor who is not good at sensing employees’ emotions and does not have a good understanding of your emotions.”

##### Unethical pro-supervisor behavior

The priming materials for unethical pro-supervisor behavior were adapted from the study by Li et al. (2022) [58]. The specific materials are as follows:

“Recently, your company received a large contract to produce new components for front-wheel-drive cars. Manager Yang made significant contributions to securing this contract, and he is likely to be promoted. The final testing of the components was completed last Friday. The first shipment begins today and runs for three weeks. Upon reviewing the test reports, you discover that the new components fail when the load speed exceeds 120% of the rated capacity. However, the manufacturer specifies that it cannot fail when loaded at 130% of rated capacity. While drivers would not lose control of their vehicles during use, the resulting damage to the cars could cost the owners thousands of dollars and harm your company’s reputation.

According to the company’s values, safety standards are of utmost importance. Any discrepancies in the safety specification must be reported urgently, a new test needs to be arranged and all production should be halted until these tests are completed. You presented the report to Manager Yang. He acknowledged the report but did not request additional testing. He says, “Let it go; we cannot afford to delay the delivery of the new parts, or we may face significant losses.”

##### Supervisor-subordinate guanxi

The supervisor-subordinate guanxi scale developed by Guo and Li (2015) [59] was selected. The scale contains three dimensions: affective, instrumental and obligatory. Since our study focused on the emotional connection between supervisors and subordinates, only the affective dimension was applied. The dimension includes eight items. Example item is: " Sometimes my supervisor and I chat like friends, occasionally making small jokes.” A 5-point Likert scale was used, with scores ranging from 1 (strongly disagree) to 5 (strongly agree). Higher scores indicate a better supervisor-subordinate guanxi. The Cronbach’s α for the scale in the study is 0.96.

##### Scenario realism testing

To ensure that a sufficiently realistic scenario was created for this study, the Realism Manipulation Test Scale developed by Chen et al. (2011) [60] was adopted. The scale contains three items. Example item is: " At some point in my career, I might face the situation described above.” A 5-point Likert scale was used, with scores ranging from 1 (strongly disagree) to 5 (strongly agree). Higher scores indicate a higher perceived realism of the scenario. The Cronbach’s α for the scale in the study is 0.75.

##### Emotional appraisal ability

Emotional appraisal ability was measured using the Emotional Appraisal Scale developed by Wong and Law (2002) [11], which comprises four items. An example item for subordinates’ emotional appraisal ability is, “I am sensitive to my supervisor’s feelings and emotions.” A 5-point Likert scale was used, with scores ranging from 1 (strongly disagree) to 5 (strongly agree). Higher scores indicate a higher emotional appraisal ability of subordinates. The Cronbach’s α for the scale in the current study is 0.77. An example item for supervisors’ emotional appraisal ability is, “My supervisor is sensitive to employees’ feelings and emotions.” A 5-point Likert scale was also used, with scores ranging from 1 (strongly disagree) to 5 (strongly agree). Higher scores indicate a higher emotional appraisal ability of supervisors. The Cronbach’s α for the scale in the study is 0.79.

The classification of subordinates’ emotional appraisal ability into high and low levels was based on their scores on the Emotional Appraisal Ability Scale, using the scale cut-point as the criterion. The questionnaire adopted a 5-point Likert scale, with 2.5 serving as the cut-off value. Scores above 2.5 were categorized as high subordinate EAA, whereas scores below 2.5 were categorized as low subordinate EAA. Based on this classification, a 2 (supervisor EAA: high vs. low) × 2 (subordinate EAA: high vs. low) experimental design was employed.

##### Control variables

Gender, age, education, Machiavellianism, and social desirability of the participants were included as control variables. Machiavellianism and social desirability have been shown to be significantly related to unethical pro-supervisor/pro-organizational behavior [61, 62]. Machiavellianism was measured using the Machiavellianism Scale, developed by Dahling et al. (2009) [63], which consists of 16 items. An example item is, “I talk to others only to gain information that benefits me.” A 5-point Likert scale was used, with scores ranging from 1 (strongly disagree) to 5 (strongly agree). Higher scores indicate higher levels of Machiavellianism. The Cronbach’s α for this scale in the study is 0.91.

Social desirability was measured using the Chinese version of the Marlowe-Crowne Social Desirability Scale (MCSD), revised by Yang(2008) [64], which consists of 8 items. This scale measures the extent to which individuals unconsciously engage in behavior that conforms to social expectations. An example item is, “Sometimes you take advantage of others.” A 5-point Likert scale was used, with scores ranging from 1 (strongly disagree) to 5 (strongly agree). Higher scores indicate higher levels of social desirability. The Cronbach’s α for this scale in the study is 0.72.

### Research results

#### Manipulation check

The results of the independent samples t-test indicated that participants in the high supervisor emotional appraisal ability group reported significantly higher levels of supervisor emotional appraisal ability (M = 4.23, SD = 0.43) compared to those in the low supervisor emotional appraisal ability group (M = 1.44, SD = 0.29), t(158) = 47.86, *p* < 0.001. This result suggests that the manipulation of supervisor emotional appraisal ability in this study was effective.

#### Hypothesis testing

This study followed the approach of Baer et al. (2021) [65] for testing hypotheses in scenario simulation research, employing independent samples t-tests and post hoc multiple comparisons to validate the research hypotheses.

The results of the independent samples t-test showed that both SSG [t(158) = 20.74, *p* < 0.001] and UPSB [t(158) = 4.75, *p* < 0.001] were significantly higher in conditions where supervisor and subordinate emotional appraisal abilities were consistent, thereby confirming hypotheses H1a and H1b. The post hoc multiple comparisons (Table 1) revealed that, in the consistent condition, the SSG (M = 4.20) for high supervisor-high subordinate emotional appraisal ability was significantly greater than the SSG (M = 3.46) for low supervisor-low subordinate emotional appraisal ability, thus supporting hypothesis H2a. Similarly, the UPSB (M = 4.03) for high supervisor-high subordinate emotional appraisal ability was significantly greater than the UPSB (M = 2.85) for low supervisor-low subordinate emotional appraisal ability, thereby supporting hypothesis H2b. In the inconsistent condition, the SSG (M = 2.00) for low supervisor-high subordinate emotional appraisal ability was significantly greater than the SSG (M = 1.68) for high supervisor-low subordinate emotional appraisal ability, which supports hypothesis H3a. Additionally, the UPSB (M = 3.22) for low supervisor-high subordinate emotional appraisal ability was significantly greater than the UPSB (M = 1.78) for high supervisor-low subordinate emotional appraisal ability, lending support to hypothesis H3b.

**Table 1:** Study 1: Consistency of supervisors’ and subordinates’ emotional appraisal ability and its relationship with SSG and UPSB

		Supervisor-Subordinate Guanxi		
		*M*	H2a: MD	H3a: MD
			VS. LL × LS	VS. HL ×LS
Congruence	HL × HS	4.20	0.74^***^	-
	LL × LS	3.46	-	-
Incongruence	LL × HS	2.00	-	0.33^***^
	HL × LS	1.68	-	-

		Unethical Pro-supervisor Behavior		
		*M*	H2b: MD	H3b: MD
			VS. LL × LS	VS. HL × LS
Congruence	HL × HS	4.03	1.19^***^	-
	LL × LS	2.85	-	-
Incongruence	LL × HS	3.22	-	1.44^***^
	HL × LS	1.78	-	-

*MD *means Mean Difference, *HL *means high supervisor emotional appraisal ability, *HS *means high subordinate emotional appraisal ability, *LL* means low supervisor emotional appraisal ability, *LS *means low subordinate emotional appraisal ability

^***^p < 0.001

**Fig. 2:**
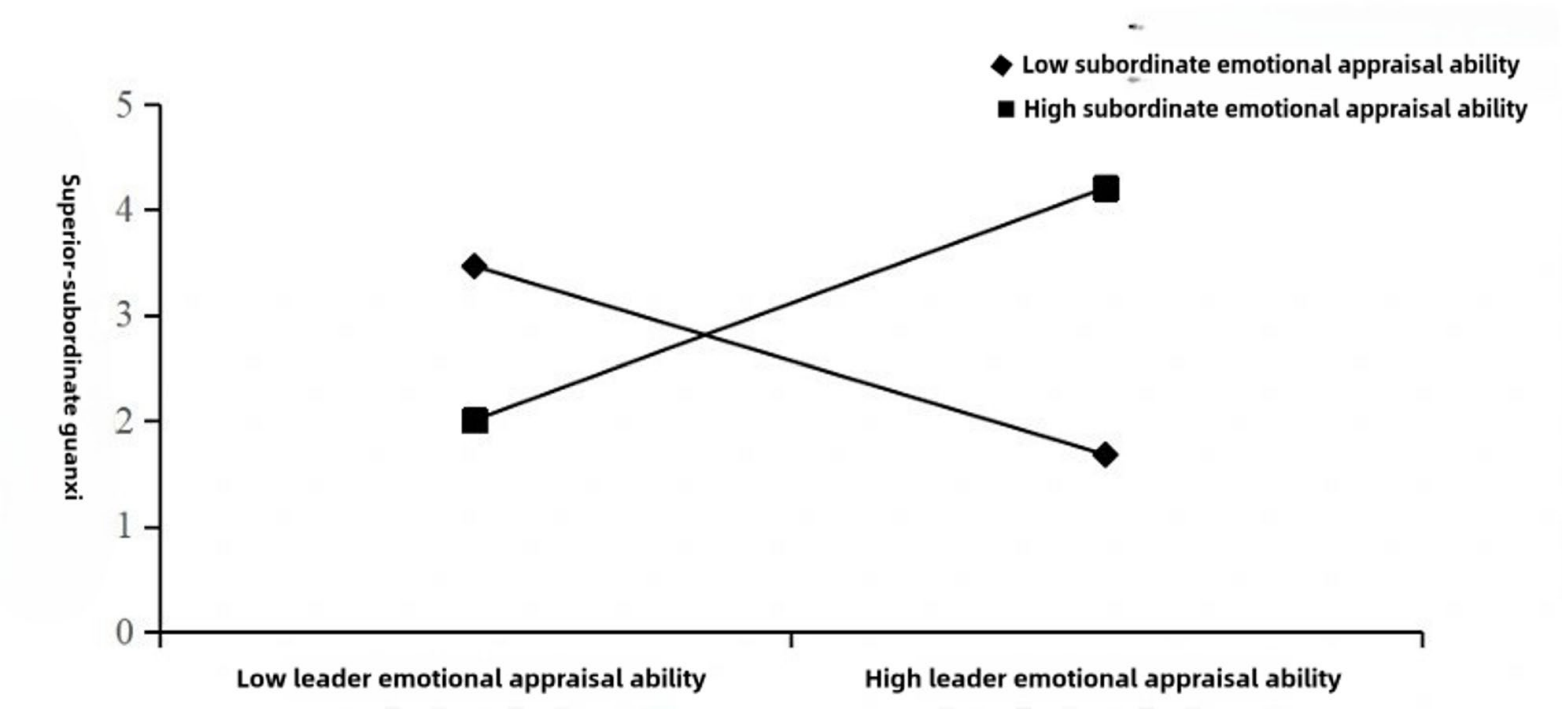
Interaction slope plot

After controlling for the control covariates, the main effect of supervisor emotional appraisal ability on the supervisor-subordinate guanxi was significant, F(1,160) = 5.22, *p* = 0.024, η²*p* = 0.03. This indicates that, compared to low supervisor emotional appraisal ability, employees are more likely to foster a better supervisor-subordinate guanxi under high supervisor emotional appraisal ability. The main effect of subordinate emotional appraisal ability on the supervisor-subordinate guanxi was also significant, F(1,160) = 30.51, *p* < 0.001, η²*p* = 0.17. This suggests that subordinates with high emotional appraisal ability are more effective in enhancing the supervisor-subordinate guanxi compared to those with low emotional appraisal ability. The interaction between supervisor and subordinate emotional appraisal abilities on the supervisor-subordinate guanxi was significant, F(1,160) = 529.65, *p* < 0.001, η²*p* = 0.78. A simple slope analysis of this interaction revealed that when the supervisor’s emotional appraisal ability is high, the supervisor-subordinate guanxi quality is significantly higher for subordinates with high emotional appraisal ability (M = 4.2, SD = 0.44) compared to those with low emotional appraisal ability (M = 1.67, SD = 0.52). Conversely, when the supervisor’s emotional appraisal ability is low, the supervisor-subordinate guanxi quality is significantly higher for subordinates with low emotional appraisal ability (M = 3.46, SD = 0.62) compared to those with high emotional appraisal ability (M = 2.00, SD = 0.57). The slope graph is shown in Fig. 2. This finding is consistent with hypothesis H1a, indicating that consistent supervisor-subordinate emotional appraisal ability more effectively enhances the quality of the supervisor-subordinate relationship compared to inconsistent emotional appraisal ability.

**Fig. 3:**
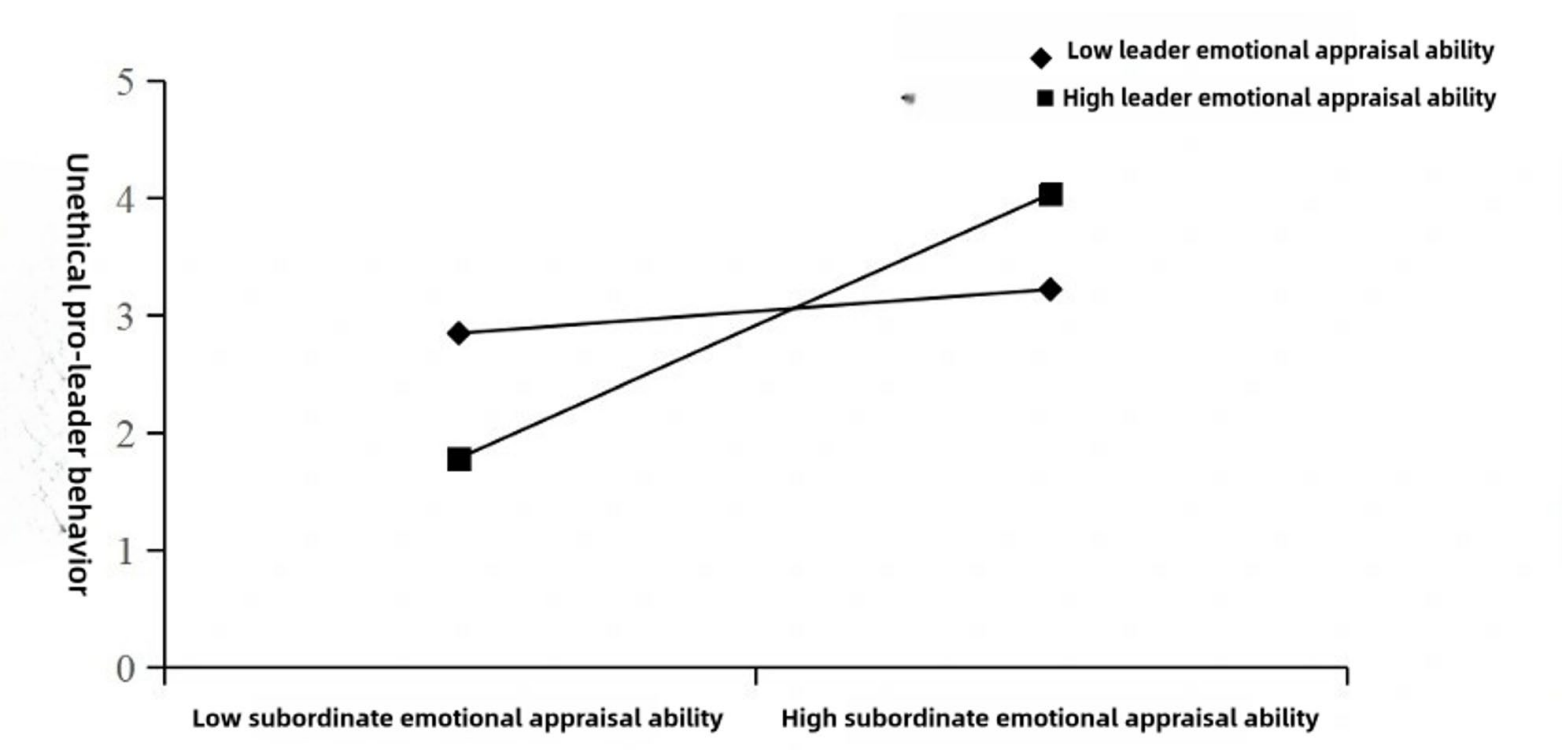
Interaction slope plot

After controlling for the control covariates, the main effect of supervisor emotional appraisal ability on UPSB was not significant, F(1,160) = 0.66, *p* = 0.42, η²*p* = 0.004. This indicates that the level of supervisor emotional appraisal ability has no significant impact on UPSB. However, the main effect of subordinate emotional appraisal ability on UPSB was significant, F(1,160) = 52.58, *p* < 0.001, η²*p* = 0.26. This suggests that subordinates with high emotional appraisal ability are more effective in promoting UPSB compared to those with low emotional appraisal ability. The interaction between supervisor and subordinate emotional appraisal ability on UPSB was significant, F(1,160) = 32.38, *p* < 0.001, η²*p* = 0.18. A simple slope analysis of this interaction showed that for subordinates with high emotional appraisal ability, UPSB under high supervisor emotional appraisal ability (M = 4.03, SD = 1.09) was significantly higher than under low supervisor emotional appraisal ability (M = 3.22, SD = 1.46). For subordinates with low emotional appraisal ability, UPSB under low supervisor emotional appraisal ability (M = 2.84, SD = 0.94) was significantly higher than under high supervisor emotional appraisal ability (M = 1.78, SD = 0.54). The slope graph is shown in Fig. 3. This finding is consistent with hypothesis H1b, indicating that consistent supervisor-subordinate emotional appraisal ability more effectively promotes subordinates’ unethical pro-supervisor behavior compared to inconsistent emotional appraisal ability.

In summary, the scenario simulation method was employed in Study 1 to examine the predictive pathways of different matching combinations on supervisor-subordinate guanxi and unethical pro-supervisor behavior among employees, demonstrating a certain degree of internal validity. However, scenario simulation is predominantly contingent upon participants’ conceptualization of the priming materials. Although the results indicate that the experimental manipulation was effective, potential discrepancies with real-world situations may still exist. To augment the ecological validity of the study, a questionnaire-based approach was adopted in Study 2 to further approximate authentic workplace conditions experienced by employees. Furthermore, Study 1 exclusively addressed a subset of the proposed hypotheses, and in Study 2, we will proceed to investigate the entirety of the theoretical hypotheses.

## Study 2: full model testing (questionnaire survey)

### Research method

#### Participants

Data were collected through the Wenjuanxing and Credamo platforms. To exclude non-employee samples, the first question was designed as, “Are you an employee of a company?” If respondents answered “No,” they were directly redirected to the survey submission page. To further verify the validity of the questionnaire, a lie detector question was set as Question 31, which stated: “Please select ‘Completely Disagree’ for this question.”

The data were collected in three waves, with three weeks separating the first and second waves and six weeks separating the second and third waves. At Time 1, employees rated demographic information, the subordinate emotional appraisal ability questionnaire, and the supervisor emotional appraisal ability questionnaire(725 responses). At Time 2, employees rated the supervisor-subordinate guanxi questionnaire (697 responses). At Time 3, 675 employees rated unethical pro-supervisor behavior questionnaire and control variables (Machiavellianism, social desirability). Multivariate analysis of variance revealed that the mean item scores of the study variables of those participants who dropped out of the study did not differ from those who remained( *p* = 0.891).

Participants were recruited using a convenience sampling method. Prior to data collection, participants were informed about the purpose and procedure of the study and provided their informed consent. Subsequently, they received a survey link hosted on a secure online platform. Participants were informed that their participation was voluntary, confidential, and anonymous, and that they could withdraw from the study at any stage. The inclusion criteria for participation were as follows: (1) participants had to be adults aged 18 years or older; (2) participants were required to be currently employed in an organization; and (3) participants needed to have at least 0.6 months of work experience. The study was approved by the institutional review board.

A total of 723 questionnaires were initially collected. Invalid responses were then excluded. These included cases with a response time of less than 100 s, an answer of “No” to the first screening question, failure on the attention-check item, or patterned responses. After data screening, 675 valid questionnaires were retained for the final analysis. The overall response rate was 93.4%. In the final sample, 68.4% of the respondents were male and 31.6% were female. Regarding age, 44.7% were under 25 years old, 42.4% were between 25 and 35 years old, 9.3% were between 35 and 45 years old, and 3.6% were 45 years old or above. In terms of education, 5.6% had a high school degree or below, 14.4% held an associate degree, 62.4% held a bachelor’s degree, and 17.6% held a master’s degree or above. Missing values were addressed using the Expectation-Maximization algorithm within the maximum likelihood estimation framework.

#### Measurement instruments

All research variables were measured using well-established scales. The English scales were translated into Chinese in accordance with Brislin’s (1980) [57] translation-backtranslation method.

Emotional appraisal ability. Emotional appraisal ability was measured using the Emotional Appraisal Scale developed by Wong and Law (2002) [11], consisting of four items. An example item for subordinates’ emotional appraisal ability is, “I am sensitive to my supervisor’s feelings and emotions.” A 5-point Likert scale was used, with scores ranging from 1 (strongly disagree) to 5 (strongly agree). Higher scores indicate a higher emotional appraisal ability of subordinates. The Cronbach’s α for the scale in this study is 0.83. An example item for supervisors’ emotional appraisal ability is, “My supervisor is sensitive to employees’ feelings and emotions.” A 5-point Likert scale was also used, with scores ranging from 1 (strongly disagree) to 5 (strongly agree). Higher scores indicate a higher emotional appraisal ability of supervisors. The Cronbach’s α for the scale in this study is 0.85.

The supervisor-subordinate guanxi scale developed by Guo and Li (2015) [59] was selected. The scale contains three dimensions: affective, instrumental and obligatory. Since our study focused on the emotional connection between supervisors and subordinates, only the affective dimension was utilized. The dimension comprised eight items. Example item is: " Sometimes my supervisor and I chat like friends, occasionally making small jokes.” A 5-point Likert scale was used, with scores ranging from 1 (strongly disagree) to 5 (strongly agree). Higher scores indicate a higher quality supervisor-subordinate guanxi. The Cronbach’s α for the scale in this study is 0.91.

Unethical pro-supervisor behavior (UPSB) was measured using a six-item scale developed by Johnson and Umphress (2019) [1]. A sample item is: “I have concealed information from others that could be harmful to my supervisor when necessary.” Responses were rated on a five-point Likert scale ranging from 1 (strongly disagree) to 5 (strongly agree). Higher scores indicated higher levels of unethical pro-supervisor behavior. This scale was completed by employees. The Cronbach’s α coefficient for this scale in the present study was 0.88.

Control Variables. Machiavellianism, and social approbation of the participants were included as control variables. Machiavellianism and social desirability have been shown to be significantly related to unethical pro-supervisor/pro-organizational behavior [61, 62]. Machiavellianism was measured using the Machiavellianism Scale developed by Dahling et al. (2009) [63], which consists of 16 items. An example item is, “I talk to others only to gain information that benefits me.” A 5-point Likert scale was used, with scores ranging from 1 (strongly disagree) to 5 (strongly agree). Higher scores indicate higher levels of Machiavellianism. The Cronbach’s α for this scale in the study is 0.92. Social desirability was measured using the Chinese version of the Marlowe-Crowne Social Desirability Scale (MCSD) revised by Yang(2008) [64], which consists of 8 items. This scale measures the extent to which individuals unconsciously engage in behavior that conforms to social expectations. An example item is, “Sometimes you take advantage of others.” A 5-point Likert scale was used, with scores ranging from 1 (strongly disagree) to 5 (strongly agree). Higher scores indicate higher levels of social desirability. The Cronbach’s α for this scale in the study is 0.80.

### Results

#### Descriptive statistical analysis

Table 2 presents the means, standard deviations, and correlation coefficients of the variables. A significant positive correlation was observed between each pair of the following variables: unethical pro-supervisor behavior, subordinate emotional appraisal ability, supervisor emotional appraisal ability, and supervisor-subordinate guanxi. This finding provides a foundational basis for the subsequent main effect and mediating effect analyses.

**Table 2:** Study 2: Descriptive statistics and intercorrelations

	M	SD	1	2	3	4	5	6
1. Supervisor emotional appraisal ability	3.70	0.81	1					
2.Subordinate emotional appraisal ability	3.72	0.79	0.65^***^	1				
3.Supervisor-subordinate guanxi	3.51	0.83	0.65^***^	0.47^***^	1			
4. Unethical pro-supervisor behavior	3.07	0.96	0.37^***^	0.67^***^	0.49^***^	1		
5. Machiavellianism	3.12	0.80	0.27^***^	0.34^***^	0.33^***^	0.74^***^	1	
6. Social approbation	3.06	0.76	0.21^***^	0.27^***^	0.26^***^	0.55^***^	0.77^***^	1

*p < 0.05, **p < 0.01, ***p < 0.001

#### Discriminant validity

Discriminant validity of the four-factor model was tested using Amos 23.0. The analysis results are shown in Table 3. Among the different factor models, the four-factor model had the best fit (χ²/df = 4.2, RMSEA = 0.07, CFI = 0.93, TLI = 0.92). In addition to the four-factor baseline model, three competing models were tested by combining the variables, but their goodness-of-fit was weaker than that of the baseline model, indicating good discriminant validity among the four variables.

**Table 3:** Results of CFA for study 2

Model	χ^2^	df	χ^2^/df	RMSEA	CFI	TLI
Four factors	852.56	203	4.2	0.07	0.93	0.92
Three factors	1070.29	206	5.20	0.08	0.90	0.89
Two factors	2368.29	208	11.39	0.12	0.75	0.72
Single factor	2824.46	209	13.51	0.14	0.70	0.67

*p < 0.05, **p < 0.01, ***p < 0.001

#### Common method bias and multicollinearity tests

First, Harman’s single-factor test was conducted to examine the issue of common method bias. The results showed that the first unrotated factor accounted for 32.42% of the total variance, which was below the critical threshold of 40%. In addition, the study further assessed common method bias using the Unmeasured Latent Method Construct(ULMC) approach. The results indicated that, compared with the original model, the differences in model fit indices after adding the method factor were all within acceptable standards. Specifically, the changes in RMSEA and SRMR did not exceed 0.05, and the changes in CFI and TLI did not exceed 0.10. These results suggest that common method bias was not a serious concern in this study.

Variance inflation factors were calculated to assess potential multicollinearity among the predictor variables. The diagnostic results showed that all VIF values ranged from 1.95 to 2.72, which were far below the commonly accepted conservative threshold (VIF < 5). This indicates that there was no multicollinearity problem in the data.

#### Hypothesis testing

Polynomial regression and response surface analysis was used to test the congruence hypothesis. Before conducting the hypothesis testing, the distribution proportions of the sample were analyzed to determine whether the response surface and polynomial regression methods were appropriate. The results showed that 58.85% of the samples had congruence between the supervisor’s emotional appraisal ability and the subordinate’s emotional appraisal ability, 20.15% had a higher supervisor emotional appraisal ability compared to the subordinate’s, and 20.00% had a lower supervisor emotional appraisal ability compared to the subordinate’s—all of which exceeded the 10% threshold. Therefore, the sample was suitable for using response surface and polynomial regression methods.

To reduce multicollinearity during the construction of product terms and to facilitate interpretation of the results, we used grand-mean-centered values of supervisor emotional appraisal ability (L) and subordinate emotional appraisal ability (P) to create the interaction term [66].

We then conducted multicollinearity diagnostics for the polynomial regression model constructed with the group-mean-centered emotional appraisal ability variables and other related predictors. The results indicated that all polynomial terms—including linear, quadratic, and interaction terms—had variance inflation factor (VIF) values ranging from 1.90 to 2.81. These values were well below the conservative threshold of 5, suggesting that multicollinearity was effectively controlled.

1. Testing the Effect of Congruence.

As shown by the results of M2 and M5 in Table 4, when the quadratic terms were added, the change in R-squared (ΔR²) was significant. This indicates that the congruence effect between the supervisor’s and subordinate’s emotional appraisal abilities exists when the dependent variables are unethical pro-supervisor behavior and the superior -subordinate guanxi, respectively.

**Table 4:** Polynomial regression analysis and response surface analysis results for Study 2

Variables	Unethical pro-supervisor behavior			Supervisor-subordinate guanxi	
	M1	M2	M3	M4	M5
constant	0.94^***^	-1.01^***^	0.31	3.18^***^	3.20^***^
Gender	− 0.02	− 0.02	0.00	0.10*	− 0.09
Age	− 0.01	− 0.00	− 0.01	0.01	0.01
Education	− 0.03	− 0.33	− 0.03	0.00	0.00
Machiavellianism	0.80^***^	0.79^***^	0.77^***^	0.08	0.07
Social approbation	− 0.04	− 0.05	− 0.05	0.02	0.02
Subordinate emotional appraisal ability(P)	0.26^***^	0.26^***^	0.18^***^	0.41^***^	0.39^***^
Supervisor emotional appraisal ability (L)	0.08^*^	0.05	− 0.03	0.36^***^	0.38^***^
P^2^		− 0.09^*^	− 0.07		− 0.11^**^
P×L		0.163^***^	0.129^**^		0.15^***^
L^2^		− 0.08^*^	− 0.07		− 0.02
Supervisor-subordinate guanxi			0.22^***^		
* R* ^*2*^	0.60^***^	0.61^***^	0.63^***^	0.54^***^	0.55^***^
* DR* ^*2*^		0.01^**^	0.02^***^		0.01^**^
Response surface analysis					
Congruence line (P = L)					
slope (b1 + b2)		0.32^***^			0.76^***^
curvature (b3 + b4 +b5)		− 0.004			0.03
Incongruence line (P=-L)					
slope (b1-b2)		0.21^*^			0.01
curvature (b3-b4 + b5)		− 0.33^***^			− 0.28^***^
Lateral displacement quantity					
(b2- b1)/[2×(b3 - b4 + b5)]		0.30^*^			0.03

*p < 0.05; **p < 0.01; ***p < 0.001

**Fig. 4:**
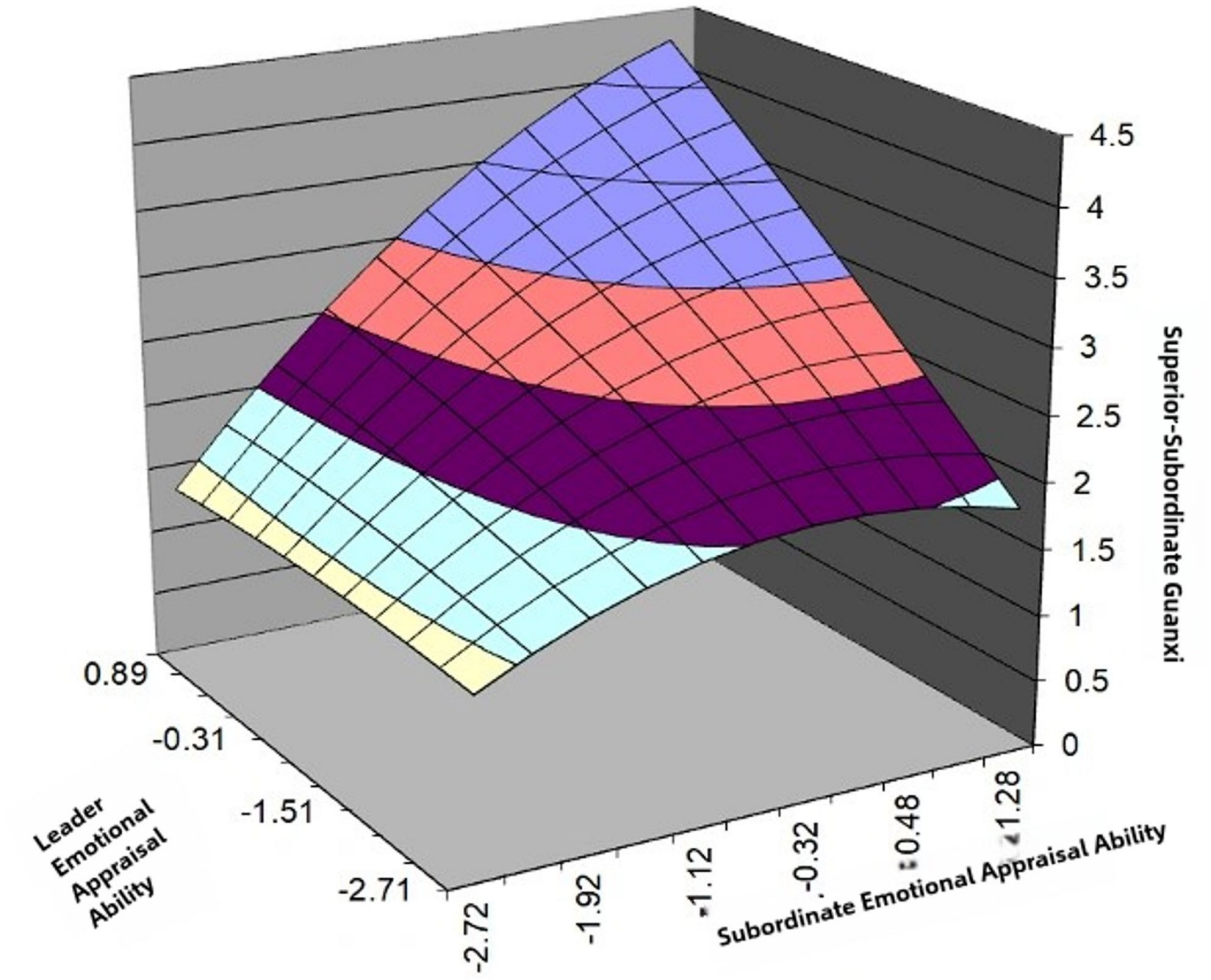
Supervisor-subordinate emotional appraisal ability (In)congruence on SSG for study 2

When the dependent variable is the supervisor-subordinate guanxi, as depicted in M5, the curvature of the incongruence line (P = -L) is negative and significant (curvature = -0.28, *p* < 0.001), thus supporting H1a. As shown in Fig. 4, the response surface is convex along the incongruence line (P = -L), indicating that the more congruent the emotional appraisal ability of the supervisor and subordinate, the better the supervisor-subordinate guanxi. Along the congruence line (P = L), the slope of the cross-section is significantly positive (slope = 0.76, *p* < 0.001), and the curvature of the response surface along the congruence line is not significant (curvature = -0.004, n.s.), suggesting that the supervisor-subordinate guanxi is better when there is high congruence between the supervisor’s and subordinate’s emotional appraisal ability compared to low congruence, thus supporting H2a. Along the incongruence line (P = -L), the slope of the cross-section is positive (slope = 0.01, n.s.) but not significant, with an offset of 0.03 (*p* > 0.05), indicating no significant difference in the supervisor-subordinate guanxi between the cases of low supervisor-high subordinate and high supervisor-low subordinate emotional appraisal ability, thereby not supporting H3a.

**Fig. 5:**
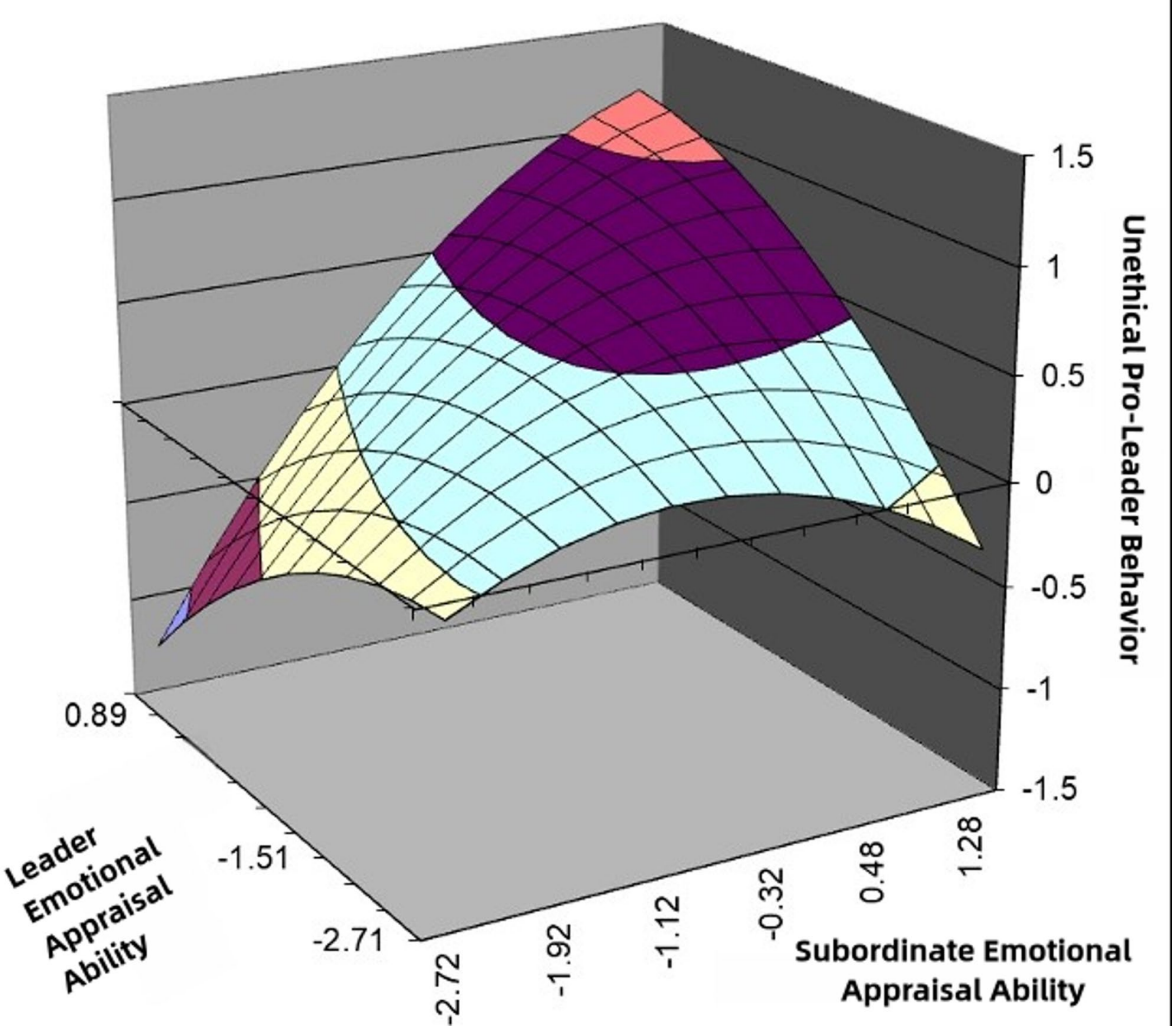
Supervisor-subordinate emotional appraisal ability (In)congruence on UPSB for study 2

When the dependent variable is unethical pro-supervisor behavior, as depicted in M2, the curvature of the incongruence line (P = -L) is negative and significant (curvature = -0.33, *p* < 0.001), supporting H1b. As shown in Fig. 5, the response surface is convex along the incongruence line (P = -L), indicating that the more congruent the emotional appraisal ability of the supervisor and subordinate, the more unethical pro-supervisor behavior occurs. Along the congruence line (P = L), the slope of the cross-section is significantly positive (slope = 0.32, *p* < 0.001), and the curvature of the response surface along the congruence line is not significant (curvature = 0.03, n.s.), suggesting that unethical pro-supervisor behavior is more frequent when there is high congruence between the supervisor’s and subordinate’s emotional appraisal ability compared to low congruence, thus supporting H2b. Along the incongruence line (P = -L), the slope of the cross-section is significantly positive (slope = 0.21, *p* < 0.05), with an offset of 0.30 (*p* < 0.05), indicating that unethical pro-supervisor behavior is more frequent in the case of low supervisor-high subordinate emotional appraisal ability incongruence than in the case of high supervisor-low subordinate incongruence, thereby supporting H3b.

(2) Mediation effect test.

The mediation effect was tested using the block variable approach proposed by Edwards and Cable (2009) [67]. We created a composite variable to represent the congruence between subordinates’ and supervisors’ emotional appraisal ability. Specifically, the block variable was generated by multiplying each polynomial term (P, L, P², PL, and L²) by its corresponding unstandardized regression coefficient obtained from the polynomial regression analysis, and then summing these weighted terms. This composite variable captured the overall congruence in emotional appraisal ability between supervisors and subordinates. Subsequently, the hypothesized mediation model was tested using Model 7 of Hayes’ PROCESS macro.

As shown in Table 5, the congruence of supervisor-subordinate emotional appraisal ability can significantly predict SSG (b = 1.04, *p* < 0.001) and UPSB (b = 0.79, *p* < 0.001) respectively. SSG has a significantly positive impact on subordinate unethical pro-supervisor behavior (b = 0.56, *p* < 0.001). Furthermore, the supervisor-subordinate emotional appraisal ability congruence influences subordinate unethical pro-supervisor behavior through SSG (b = 0.35, *p* < 0.001), with a 95% CI = [0.23, 0.49], where the confidence interval does not include 0, indicating that the mediation effect of SSG is significant, thereby supporting hypothesis H4.

**Table 5:** Test of mediating effects for study 2

Variables	Mediating variable	Dependent variable
	Supervisor-subordinate guanxi	Unethical pro-supervisor behavior
The matching of supervisor-subordinate emotional appraisal ability(Block)	1.04^***^(0.04)	0.79^***^(0.06)
Supervisor-subordinate guanxi Sex	− 0.10^*^(0.05)	0.56^***^(0.04)
Sex×Block	0.01(0.08)	
Mediation Path Effect		
Mediation Pathway	Effect size	95% CI
Matching→SSG→UPSB	0.35^***^	[0.23, 0.49]
Indirect effects of gender	0.004	[− 0.06, 0.07]

*N* = 675, The coefficients in the table are unstandardized coefficients (with standard errors in parentheses), ^*^p < 0.05, ^**^p < 0.01, ^***^p < 0.001

(3) Moderated mediation effect test.

The moderating effect of gender was further tested by using model 7 in the PROCESS macro program. The results showed that the moderating effect of gender on the relationship between supervisor-subordinate emotional appraisal ability congruence and SSG was not significant (b = 0.01, *p* = 0.9), with a 95% CI = [-0.16, 0.18], that includes 0. The moderated mediation effect was also not significant (b = 0.004, *p* = 0.8), with a 95% CI = [-0.06, 0.07], that includes 0. Thus, hypothesis 5 was not supported.

## Discussion

### Results and analysis

A combination of scenario simulation experiments and questionnaires was used to test the hypothesized model. The results supported most of the research hypotheses, although a few were not confirmed.

First, regarding the impact of incongruence in supervisor-subordinate emotional appraisals ability on their relationship, Studies 1 and 2 yielded discrepant findings. Study 1 (scenario experiment) demonstrated that supervisor-subordinate guanxi was significantly higher when the subordinate’s emotional appraisal ability was higher than the supervisor’s, compared to the opposite scenario. However, this asymmetry effect was not replicated in Study 2 (questionnaire survey). One plausible account for this divergence resides in the methodological distinctions between the two investigations. Study 1 used a controlled experimental design with manipulated scenarios, which may have heightened participants’ attention to the supervisor’s emotional cues and their own behavioral intentions. In contrast, Study 2 captured naturally occurring workplace dynamics over time, where contextual noise (e.g., organizational culture, task complexity, or competing demands) may have attenuated the effect of incongruence in supervisor-subordinate emotional appraisal ability on SSG. Therefore, the nonsignificant finding in Study 2 suggests that the asymmetric incongruence effect on supervisor–subordinate guanxi may be context-dependent. Future research could further examine the boundary conditions under which this asymmetry effect emerges.

Second, this study did not find a significant moderating effect of gender, which is inconsistent with the hypothesis. This result can be explained from two perspectives. On the one hand, it may be reasonably interpreted from the perspective that individual behavior is strongly shaped by the social norms of the context in which it occurs [68–72]. Social norms refer to the shared expectations among group members regarding appropriate behavior in specific situations. They guide individual behavior through mechanisms such as internalization and social learning [68, 69]. In the specific social context of “supervisor–subordinate interactions” examined in this study, there exist clear and powerful occupational norms that regulate workplace behavior [71]. These norms require subordinates—regardless of gender—to demonstrate professionalism, reliability, and cooperativeness in their interactions. Within such a strong normative context, individuals tend to align their behaviors with situational expectations in order to adapt to the environment and maintain a positive social image [69, 72]. Accordingly, both male and female subordinates in this study may have internalized and adhered to a common behavioral repertoire associated with the subordinate role, resulting in a high degree of congruence in emotional appraisal ability and interpersonal responses. Consequently, the potential behavioral differences arising from broader gender role orientations might have been overshadowed by the unifying influence of workplace normative expectations, thereby leading to the nonsignificant moderating effect of gender observed in this study. On the other hand, this nonsignificant result may indicate that gender does not play a meaningful moderating role in the process of supervisor–subordinate emotional appraisal ability congruence. One possible explanation is that the binary measurement of gender (male vs. female) may lack the sensitivity required to capture gender-related psychological differences. Prior research suggests that psychological gender orientation—such as instrumentality (masculine traits) versus expressiveness (feminine traits)—may be a better predictor of emotional attunement and relational behavior than biological sex alone [73]. It is possible that within-gender variation in gender role identity moderates the effects of EAA congruence. Future research may benefit from incorporating more nuanced measures of gender role identity.

### Research implications

Drawing on the findings of this study, several theoretical contributions can be highlighted: First, the study extends the understanding of the effects of supervisor-subordinate congruence, particularly in terms of its potential negative effects. Previous research on the congruence of positive attributes has confirmed that congruence between supervisors and subordinates can lead to positive outcomes. For example, Weber and Avey (2019) [74] found that value congruence between supervisors and subordinates enhances employees’ organizational commitment. Similarly, Deng et al. (2023) [75] reported that such congruence strengthens subordinates’perceived organizational status. Atalla et al. (2024) further showed that perceived value congruence is negatively associated with subordinates’ organizational deviance [76]. In addition, Shaw and Mao (2023) [77] found that supervisor–subordinate congruence in humility promotes subordinates’ voice behavior. However, the results of the present study revealed that congruence in emotional appraisal ability between supervisors and subordinates may also lead to a negative consequence—unethical pro-supervisor behavior. This finding extends the current understanding of the effects of supervisor–subordinate congruence effects on employee behavior by suggesting that congruence in positive attributes can be a double-edged sword: it can generate both beneficial and detrimental outcomes. Therefore, this study advances the existing theory of supervisor–subordinate congruence by highlighting its complex and bidirectional nature, and it calls for future research to examine this phenomenon from multiple theoretical perspectives.

Second, this study advances the research on the dark side of emotional intelligence by shifting the focus from the individual cognitive level to the dyadic interaction process within supervisor–subordinate relationships. Recent studies have begun to recognize that emotional intelligence does not always lead to positive outcomes. An increasing body of literature has identified its dark side, such as heightened stress reactivity, emotional manipulation, deceptive behavior, and antisocial tendencies [78–82]. However, most of these studies have focused primarily on the individual level, leaving the interpersonal dynamics of emotional intelligence, particularly within vertical power relationships, largely unexplored. By uncovering how the emotional consensus formed when both supervisors and subordinates possess high levels of emotional appraisal ability can trigger unethical behaviors, this study extends the understanding of emotional intelligence’s dark side from an individual cognitive perspective to a dyadic interaction perspective. This not only reinforces the conceptual complexity of emotional intelligence but also provides a more nuanced explanation of how its detrimental effects unfold within dyadic relationships.

Finally, this study examined the mediating mechanism of supervisor-subordinate guanxi within the theoretical framework of ability congruence. This approach reveals a paradigm shift in understanding the antecedents of relationship construction—from unilateral attributes to dyadic characteristics. Supervisor-subordinate guanxi serve as a critical bridge linking supervisor characteristics to subordinate behaviors, and its mediating role has been well established in prior research [83–88]. However, most existing studies have predominantly focused on either supervisors’ personal traits or subordinates’ individual perceptions when predicting relationship quality, thereby overlooking the inherently interactive nature of relationships. By investigating superior–subordinate congruence in emotional appraisal ability as a dyadic antecedent of relationship quality, this study reconceptualizes the supervisor-subordinate guanxi as a dynamic system shaped by the mutual fit between both parties.

In practical terms, this study found that high–high congruence in supervisors’ and subordinates’ emotional appraisal ability can strengthen superior–subordinate guanxi, thereby increasing the risk of unethical pro-supervisor behavior. Based on the risk characteristics of this congruence effect, several practical implications are proposed to help organizations mitigate the occurrence of UPSB.

First, organizations should develop differentiated ethical climate intervention mechanisms. For teams characterized by high–high EAA congruence, regular ethical scenario-based training should be conducted to enhance employees’ ability to identify and resist unethical requests. In addition, cross-departmental ethics supervision committees should be established to routinely monitor the decision-making processes of these teams. Such oversight can help neutralize the hidden breeding ground for unethical conduct that may emerge from high mutual emotional insight and interpersonal synchrony between supervisors and subordinates.

Second, organizations should establish formal issue-escalation channels that bypass loyalty-based constraints. Independent reporting mechanisms, such as cross-level anonymous reporting systems or third-party ethics review committees, should be implemented. These channels must clearly stipulate that employees who refuse or report unethical pro-supervisor behaviors can safely escalate such issues to higher levels or independent bodies without fear of retaliation. This institutional design aims to break the loyalty-induced alliance barrier that may form under high–high EAA congruence due to strong emotional resonance, thus preventing employees from engaging in unethical acts out of loyalty or interpersonal pressure.

Finally, organizations should activate mechanisms to alleviate reciprocity pressure stemming from supervisor performance metrics. Supervisors’ KPIs should include anti-short-termism components by incorporating indicators such as the team’s incidence of unethical behavior into performance evaluations and linking them directly to supervisors’ compensation and promotion outcomes. At the same time, emphasis on short-term performance goals on short-term performance goals should be reduced to discourage supervisors from leveraging high EAA congruence to exert reciprocal pressure on subordinates in pursuit of unethical or short-sighted objectives.

### Limitations and future prospects

First, this study examined only the impact of congruence in the positive trait of emotional appraisal ability on subordinates’ UPSB, without considering the influence of consistency in negative traits. Specifically, if supervisors and subordinates show congruence in negative traits, such as dark personality characteristics, would this also influence subordinates’ UPSB? Should organizations pay more attention to personnel matching based on such research findings? Might there even be a need to adjust relevant personnel matching strategies? Future research could explore this direction further. Second, as unethical behavior continues to emerge within organizations, managers are increasingly recognizing the severity of this problem, making it crucial to establish relevant reward and punishment mechanisms. How effective are the organizational climate and related regulations in curbing unethical behavior? What are the respective effect sizes of rewards and punishments? Currently, little research has examined this perspective, making these questions a potential direction for future studies. Third, the field survey is still limited in its causal interpretation even though the design used is time-lag. Therefore, future research could adopt longitudinal tracking methods, such as experience sampling, to conduct repeated sampling of the same subjects at different times during workdays. This approach would allow for a deeper exploration of the dynamic relationships between variables. Additionally, there are areas for improvement in the the manipulation of variables in the experimental design of this study. Specifically, in the experimental scenario of Study 2, the phrase “you get along well with each other” conceptually overlaps to some extent with the construct of superior–subordinate guanxi to some extent. Although this conceptual overlap was balanced across all experimental conditions, future experimental research should employ more conceptually distinct and precise manipulation materials to ensure construct purity. Moreover, Study 2 relied entirely on self-report data from subordinates. Although statistical tests (Harman’s single-factor test and the ULMC approach) did not indicate severe bias, the potential influence of common method bias cannot be completely ruled out. Therefore, future research is recommended to adopt multi-source data to address this limitation. Finally, the age distribution of participants in this study was skewed toward younger employees, with 87.1% of the sample being under the age of 35. The findings may not be directly generalizable to older employees or those at different stages of their career development. Future research should test the proposed model using more demographically diverse samples to more comprehensively evaluate its external validity, particularly in terms of age, tenure, and hierarchical position.

## Data Availability

The datasets generated during and/or analyzed during the current study are available from the corresponding author on reasonable request.

## Abbreviations

UPSB: Unethical Pro-supervisor Behavior
SSG: Supervisor-Subordinate Guanxi
LMX: Leader-Member Exchange
EAA: Emotional Appraisal Ability
